# Telmisartan Is a Promising Agent for Managing Neuropathic Pain and Delaying Opioid Analgesic Tolerance in Rats

**DOI:** 10.3390/ijms24097970

**Published:** 2023-04-27

**Authors:** David Á. Karádi, Anna Rita Galambos, Péter P. Lakatos, Joost Apenberg, Sarah K. Abbood, Mihály Balogh, Kornél Király, Pál Riba, Nariman Essmat, Edina Szűcs, Sándor Benyhe, Zoltán V. Varga, Éva Szökő, Tamás Tábi, Mahmoud Al-Khrasani

**Affiliations:** 1 Department of Pharmacology and Pharmacotherapy, Faculty of Medicine, Semmelweis University, Nagyvárad tér 4, H-1089 Budapest, Hungary; karadi.david_arpad@med.semmelweis-univ.hu (D.Á.K.); galambos.anna@phd.semmelweis.hu (A.R.G.); semmelweis.joost@gmail.com (J.A.); abbood.sarah@phd.semmelweis.hu (S.K.A.); balogh.mihaly@med.semmelweis-univ.hu (M.B.); kiraly.kornel@med.semmelweis-univ.hu (K.K.); riba.pal@med.semmelweis-univ.hu (P.R.); nariman.gomaa@phd.semmelweis.hu (N.E.); varga.zoltan@med.semmelweis-univ.hu (Z.V.V.); 2 Department of Pharmacodynamics, Faculty of Pharmacy, Semmelweis University, Nagyvárad tér 4, H-1089 Budapest, Hungary; lakatos.peter@pharma.semmelweis-univ.hu (P.P.L.); szoko.eva@pharma.semmelweis-univ.hu (É.S.); tabi.tamas@pharma.semmelweis-univ.hu (T.T.); 3 Pharmaceutical Analysis, Groningen Research Institute of Pharmacy, Faculty of Science and Engineering, University of Groningen, 9700 AD Groningen, The Netherlands; 4 Biological Research Center, Institute of Biochemistry, Temesvári krt. 62, H-6726 Szeged, Hungary; szucs.edina@brc.hu (E.S.); benyhe.sandor@brc.hu (S.B.)

**Keywords:** neuropathic pain, neuropathy, opioids, opioid tolerance, morphine, RAS, angiotensin receptor type 1, telmisartan, losartan

## Abstract

Despite the large arsenal of analgesic medications, neuropathic pain (NP) management is not solved yet. Angiotensin II receptor type 1 (AT1) has been identified as a potential target in NP therapy. Here, we investigate the antiallodynic effect of AT1 blockers telmisartan and losartan, and particularly their combination with morphine on rat mononeuropathic pain following acute or chronic oral administration. The impact of telmisartan on morphine analgesic tolerance was also assessed using the rat tail-flick assay. Morphine potency and efficacy in spinal cord samples of treated neuropathic animals were assessed by [^35^S]GTPγS-binding assay. Finally, the glutamate content of the cerebrospinal fluid (CSF) was measured by capillary electrophoresis. Oral telmisartan or losartan in higher doses showed an acute antiallodynic effect. In the chronic treatment study, the combination of subanalgesic doses of telmisartan and morphine ameliorated allodynia and resulted in a leftward shift in the dose–response curve of morphine in the [^35^S]GTPγS binding assay and increased CSF glutamate content. Telmisartan delayed morphine analgesic-tolerance development. Our study has identified a promising combination therapy composed of telmisartan and morphine for NP and opioid tolerance. Since telmisartan is an inhibitor of AT1 and activator of PPAR-γ, future studies are needed to analyze the effect of each component.

## 1. Introduction

Management of neuropathic pain (NP) remains a major challenge for clinicians as it affects 7–10% of the general population [1,2,3]; the effectiveness of currently available drugs is not nearly satisfactory and the treatment is often associated with risk of unwanted side effects [4,5]. Several guidelines are in agreement that the first-line medications in the treatment of NP are tricyclic antidepressants (TCAs), serotonin and norepinephrine reuptake inhibitors (SNRIs) or gabapentinoids [4,6,7]. With respect to effectiveness, the number needed to treat (NNT) values of these agents remain high [5]; moreover, they lack prompt onset of action [8,9]. Therefore, vast efforts are being made in order to identify novel pharmacological targets or to reposition already marketed drugs that could be incorporated into the treatment regimens of NP.

Opioid analgesics are the mainstay of acute and chronic pain management [10,11,12]; however, their use is hampered by their side effects and the development of analgesic tolerance through long-term administration [11]. The effectiveness of opioids in NP remains controversial [13,14]. In the past, it has been shown that a decreased number or G protein coupling of spinal µ-opioid receptors in neuropathy may be responsible for the loss of opioid efficacy [14,15,16,17]. In this regard, a decrease in the functional µ-opioid receptor reserve may seem similar to that seen in opioid analgesic tolerance [18].

Since the discovery of the renin–angiotensin system (RAS), it has become a highly exploitable pharmacological target in the management of cardiovascular diseases [19,20]. RAS inhibitors are widely available, cheap, safe and well-tolerated drugs, making them ideal candidates for drug repurposing efforts. The role of angiotensin receptors and ligands in the central nervous system (CNS) and their possible therapeutic use in the alleviation of NP, among other pain types, are emerging as an important area of pain research. Other groups and we ourselves have reported on the connection between the RAS and NP [5,21,22] and possible pharmacological interactions between the opioid and angiotensin systems regarding analgesia [23]. In the present work, we provide, to the best of our knowledge, the first evidence for enhanced analgesia following co-administration of a clinically, widely used, angiotensin receptor blocker (ARB) and morphine in a neuropathic rodent model. In addition, we demonstrate that the ARB telmisartan reduces the development of opioid analgesic tolerance during chronic morphine treatment.

## 2. Results

### 2.1. Telmisartan or Losartan Produces Acute Antiallodynic Effect in Neuropathic Pain Evoked by Sciatic Nerve Injury

**Figure 1:**
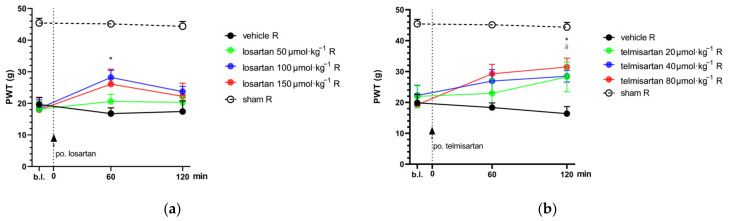
The antiallodynic effect of losartan (**a**) and telmisartan (**b**) following acute oral administration via orogastric feeding tube. Means of PWT ± S.E.M. are depicted in grams, obtained from the animals’ right (operated; R) hind paws on the 14th day after pSNL at the indicated time points. Data obtained from the intact (left) hind paws of the animals are excluded for better visual clarity; however, they are presented in Appendix A (Figure A1). Abbreviation “b.l.” stands for baseline. Panel (**a**) * *p* < 0.05 between losartan 100 µmol·kg^−1^ BW and vehicle, two-way ANOVA followed by Tukey’s post hoc test, *n* = 5–9 per group. F (time; 1.925, 51.98) = 6.607, *p* = 0.0031. F (treatment group; 4, 27) = 19.44, *p* < 0.0001. Panel (**b**) * *p* < 0.05 between telmisartan 80 µmol·kg^−1^ BW and vehicle, ^δ^ *p* < 0.05 between telmisartan 40 µmol·kg^−1^ BW and vehicle, two-way ANOVA followed by Tukey’s post hoc test, *n* = 5–11 per group. F (time; 1.847, 51.72) = 7.903 *p* = 0.0013. F (treatment group; 4, 28) = 12.32 *p* < 0.0001.

The acute effect of orally administered losartan (50, 100 and 150 µmol·kg^−1^ body weight (BW)) and telmisartan (20, 40 and 80 µmol·kg^−1^ BW) was assessed by dynamic plantar esthesiometry (DPA) two weeks following peripheral nerve injury, induced by partial sciatic nerve ligation (pSNL), in animals showing significant allodynia, the hallmark symptom of neuropathic pain (Figure 1).

Telmisartan at the two higher test doses (40 and 80 µmol·kg^−1^ BW) showed a significant antiallodynic effect 2 h after systemic (oral) administration when compared to its vehicle, 1% HEC (paw withdrawal threshold (PWT): 28.47 ± 1.91, *n* = 9 vs. 16.35 ± 2.30, *n* = 6; *p* = 0.013 and 31.48 ± 2.83, *n* = 6 vs. 16.35 ± 2.30, *n* = 6; *p* = 0.014, respectively). Losartan in doses of 100 or 150 µmol·kg^−1^ BW produced moderate antiallodynia with a peak effect at 60 min after systemic administration; however, significance was achieved only following the 100 µmol·kg^−1^ BW dose compared to the vehicle treated group (PWT: 28.19 ± 2.25, *n* = 9 vs. 16.76 ± 1.80, *n* = 5; *p* = 0.013) (Figure 1).

### 2.2. Combination of Telmisartan and Morphine in Subanalgesic Doses Alleviates Neuropathic Pain Evoked by Sciatic Nerve Injury

**Figure 2:**
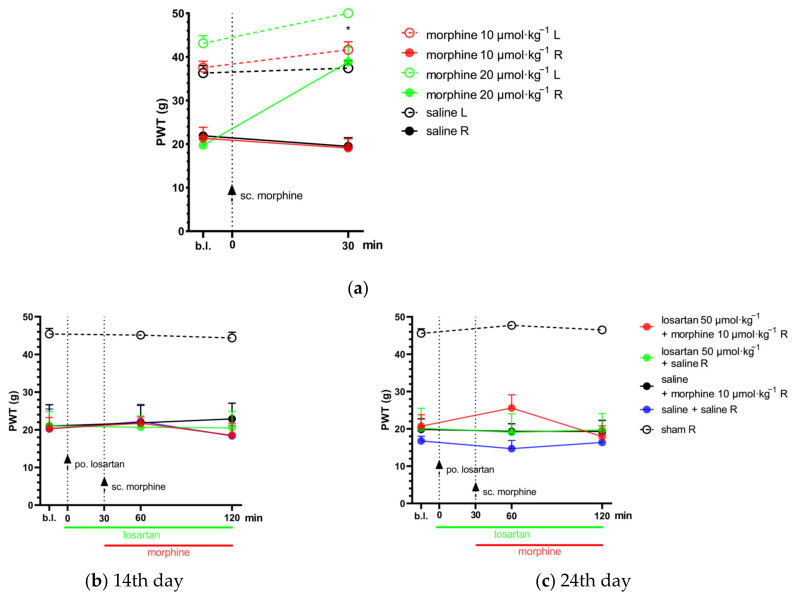

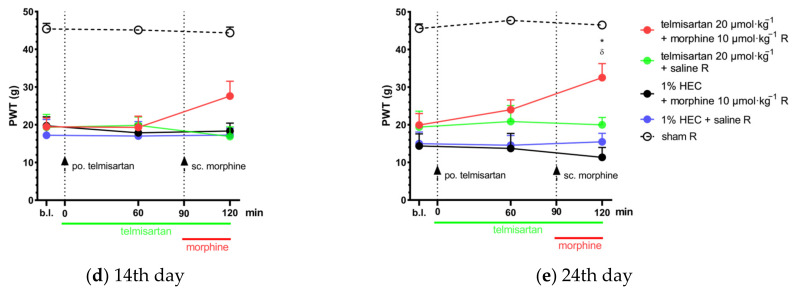
(**a**) Determination of the acute antiallodynic effect of subcutaneous morphine at doses of 10 and 20 µmol·kg^−1^ BW. The antiallodynic effect was tested at 30 min, which corresponds to the peak effect of morphine [14,24]. * *p* < 0.05 versus vehicle, two-way ANOVA followed by Tukey’s post hoc test, *n* = 5–12 per group. F (time; 1, 38) = 13.19, *p* = 0.0008. F (treatment group; 5, 38) = 38.06, *p* < 0.0001. (**b**,**c**) The antiallodynic effect of po. losartan, sc. morphine and their combination, following acute and chronic treatment on the 14th and 24th day post-pSNL, respectively. Abbreviation “b.l.” stands for baseline. Two-way ANOVA followed by Tukey’s post hoc test, *n* = 5–6 per group. 14th day: F (time; 1.817, 38.15) = 0.75 *p* = 0.47; F (treatment group; 4, 21) = 12.93, *p* < 0.0001. 24th day: F (time; 1.57, 33) = 1.1, *p* = 0.33; F (treatment group; 4, 21) = 23.87, *p* < 0.0001. (**d**,**e**) The antiallodynic effect of po. telmisartan, sc. morphine and their combination, following acute and chronic treatment on the 14th and 24th day post-pSNL, respectively. * *p* < 0.05 versus vehicle, ^δ^ *P* < 0.05 vs. 1% HEC + morphine 10 µmol·kg^−1^ R, two-way ANOVA followed by Tukey’s post hoc test, *n* = 4–6 per group; 14th day: F (time; 1.711, 35.94) = 0.7 *p* = 0.48; F (treatment group; 4, 21) = 27.15, *p* < 0.0001; 24th day: F (time; 1.855, 38.96) = 4.24, *p* = 0.024; F (treatment group; 4, 21) = 27.06, *p* < 0.0001. Results are presented as mean ± S.E.M.

In this phase of the study, the acute effect of a combination of telmisartan or losartan and morphine was investigated in animals showing allodynia two weeks after pSNL. As stated above, acute treatment with losartan and telmisartan at doses of 50 and 20 µmol·kg^−1^ BW, respectively, did not cause significant analgesic effect (Figure 1). Alleviation of neuropathic pain by morphine alone was also assessed (Figure 2a) following subcutaneous administration, in order to determine the dose of morphine used as part of the combination treatments. In this set of experiments, morphine failed to alleviate neuropathic pain at a dose of 10 µmol·kg^−1^, which was chosen for further experiments as the subanalgesic dose.

The combination of telmisartan and morphine in subanalgesic doses showed a trend but no significant effect towards the alleviation of neuropathic pain after acute administration. However, following 10 days of chronic treatment, this combination was able to alleviate NP in the operated paws. Namely, at the peak-effect of the compounds, a significantly higher PWT of the operated paws was observed in the combination group when compared with the group treated with their vehicles (PWT: 32.53 ± 3.71, *n* = 6 vs. 15.46 ± 2.27, *n* = 4; *p* = 0.028) or 1% HEC plus morphine (PWT: 32.53 ± 3.71, *n* = 6 vs. 11.35 ± 2.63, *n* = 5; *p* = 0.008) (Figure 2d,e). On the other hand, the combination of subanalgesic doses of losartan and morphine failed to alleviate NP either on the 1st day of treatment (acute effect) or after 10 days of chronic administration (Figure 2b,c).

### 2.3. Impact of Chronic Telmisartan and Morphine Combination Treatment on Morphine-Stimulated [^35^S]GTPγS Binding in Spinal Cord Membranes of Neuropathic Rats

**Figure 3:**
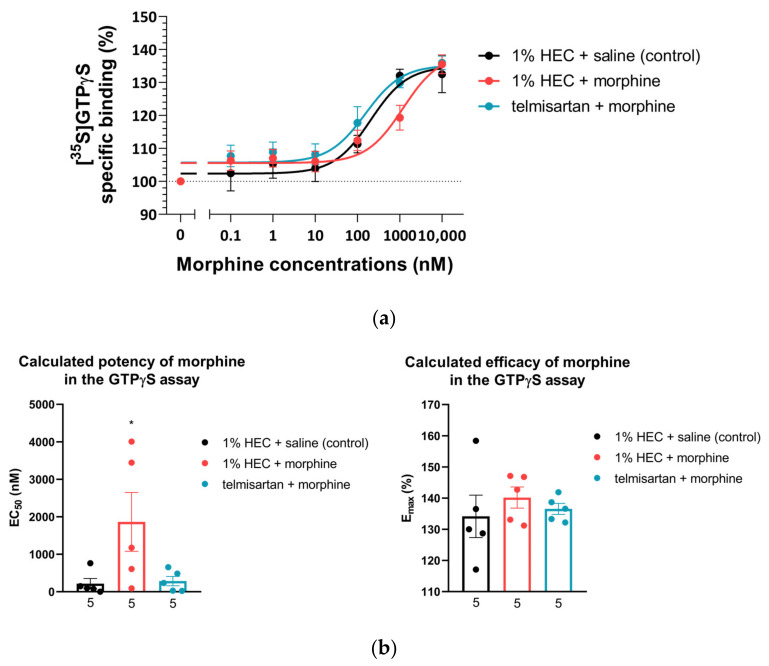
(**a**) Morphine-induced µ-opioid receptor G-protein activity on L4–L6 spinal cord membrane homogenates obtained from animals that had previously undergone 10 days of chronic treatment according to the indicated groups. *n* = 5 per group. (**b**) Calculated EC_50_ and E_max_ of morphine from the morphine-stimulated [^35^S]GTPγS binding assay on L4–L6 spinal cord samples obtained from animals that had previously undergone 10 days of chronic treatment according to the indicated groups. * *p* < 0.05 vs. all other groups, one-way ANOVA followed by Fisher’s LSD post hoc test, *n* = 5 per group. EC_50_: F (2, 12) = 4.017, *p* = 0.0462 E_max_: F (2, 12) = 0.4538, *p* = 0.6457. EC_50_ and E_max_ values were calculated individually for each animal and means ± S.E.M. are presented here.

Specific G-protein coupling of spinal µ-opioid receptors was measured using morphine-stimulated [^35^S]GTPγS binding assay and the acquired binding data are summarized in Table 1. Morphine showed similar efficacy (E_max_) for G-protein coupling in the spinal cord tissues of animals from either treatment group. On the other hand, the potency of morphine (EC_50_) with respect to [^35^S]GTPγS binding was significantly increased in spinal tissue samples obtained from animals treated chronically with the combination composed of the subanalgesic doses of telmisartan and morphine compared to the morphine-treated group (1% HEC plus morphine) (Figure 3a,b). This reduction in the [^35^S]GTPγS specific binding potency of morphine can also be observed as a rightward-shift of the morphine concentration–response curve in the indicated group (Figure 3a).

### 2.4. Telmisartan Delays the Development of Morphine Analgesic Tolerance in Rat Tail-Flick Assay

**Figure 4:**
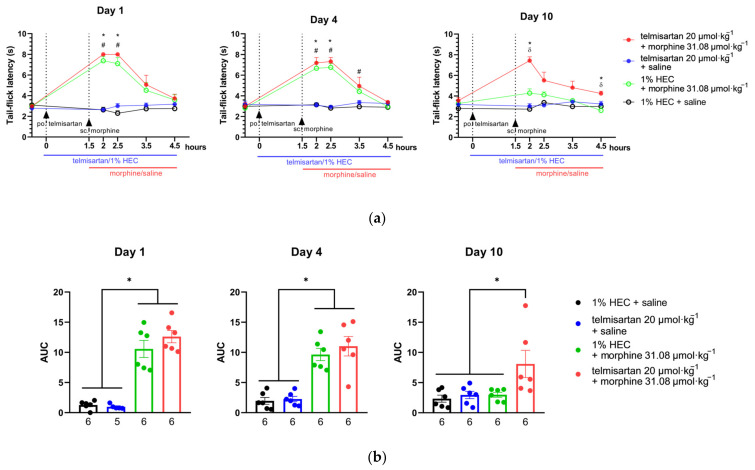
(**a**) The effect of morphine, telmisartan and their combination on the tail-flick latency of *naïve* rats (day 1) and following 4 or 10 days of chronic treatment with the indicated combinations. Telmisartan or 1% HEC was administered orally at 0 min, while morphine or saline was administered subcutaneously at 90 min to ensure that the peak effect of the two compounds coincided in time. * *p* < 0.05 between telmisartan + morphine and 1% HEC + saline, ^#^ *p* < 0.05 between 1% HEC + morphine and 1% HEC + saline, ^δ^ *p* < 0.05 between telmisartan + morphine and 1% HEC + morphine, two-way ANOVA followed by Tukey’s post hoc test, *n* = 6 per group. Day 1: F (time; 2.39, 47.79) = 30.56, *p* < 0.0001; F (treatment group; 3, 20) = 55.15, *p* < 0.0001. Day 4: F (time; 3.07, 61.34) = 34.88 *p* < 0.0001; F (treatment group; 3, 20) = 27.02, *p* < 0.0001. Day 10: F (time; 3.03, 60.63) = 9.79, *p* < 0.0001; F (treatment group; 3, 20) = 22.45, *p* < 0.0001. (**b**) Area under the curve (AUC) values calculated individually for each animal of the elapsed time—tail-flick latency curves. For each animal, the tail-flick latency value prior to treatment administration was used as the baseline for calculating AUC values. * *p* < 0.05 between indicated groups, one-way ANOVA followed by Tukey’s post hoc test, *n* = 5–6 per group, exact group sizes are shown in each graph. Day 1: F (3, 19) = 43.3, *p* < 0.0001. Day 4: F (3, 20) = 21.98, *p* < 0.0001. Day 10: F (3, 20) = 4.818, *p* = 0.011. ROUT analysis, with a Q value = 0.5% identified one outlier in the telmisartan + saline group on Day 1. Data are presented as mean ± S.E.M.

Subcutaneous morphine (31.08 µmol·kg^−1^ BW) showed a significant antinociceptive effect indicated by increased latency in the tail-flick assay of rats after acute administration (1st day). This effect peaked at 30 min following morphine administration and was retained when combined either with telmisartan or its vehicle (Figure 4). On the other hand, telmisartan (20 µmol·kg^−1^ BW) alone did not show antinociception. Animals in the studied treatment groups showed a similar response on the 4th day of chronic treatment as on the 1st (Figure 4). On the 10th day of treatment, the antinociceptive effect of morphine disappeared and did not significantly differ from the control at any time point, indicating the development of antinociceptive tolerance to morphine. However, when morphine was combined with oral telmisartan, a significant antinociception was maintained on the 4th and 10th day as well, peaking at 30 min and lasting up to 3 h (Figure 4a,b).

### 2.5. Impact of Angiotensin Receptor Blockers and Morphine on the L-Glutamate Content of the CSF

**Figure 5:**
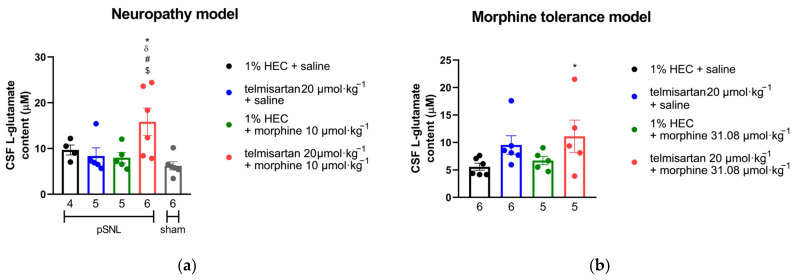
(**a**) L-glutamate content in the CSF of mononeuropathic (pSNL) and sham-operated animals following 10 days of chronic treatment with orally administered telmisartan or 1% HEC plus subcutaneously administered morphine or saline. * *p* < 0.05 vs. 1% HEC + saline, ^#^ *p* < 0.05 vs. telmisartan + saline, ^δ^ *p* < 0.05 vs. 1% HEC + morphine, ^$^ *p* < 0.05 vs. sham, one-way ANOVA followed by Fisher’s LSD post hoc test, *n* = 4–6 per group, exact group sizes are shown in each graph. F (4, 21) = 4.238, *p* = 0.0114. (**b**) L-glutamate content in the CSF of animals following 10 days of treatment according to our morphine analgesic-tolerance protocol. * *p* < 0.05 vs. 1% HEC + saline, one-way ANOVA followed by Fisher’s LSD post hoc test, *n* = 5–6 per group, exact group sizes are shown in each graph. F (3, 18) = 2.23, *p* = 0.1198.

In both in vivo models described above, cerebrospinal fluid (CSF) samples were collected from chronically treated animals and their L-glutamate content was determined by capillary electrophoresis. Based on samples from both mononeuropathic and morphine-tolerant animals, only the combination of telmisartan and morphine significantly increased CSF L-glutamate content. Individual components of the combination failed to do so in either model. (Figure 5).

### 2.6. Impact of Efficient Test Compounds on the Motor Coordination of Rats

**Figure 6:**
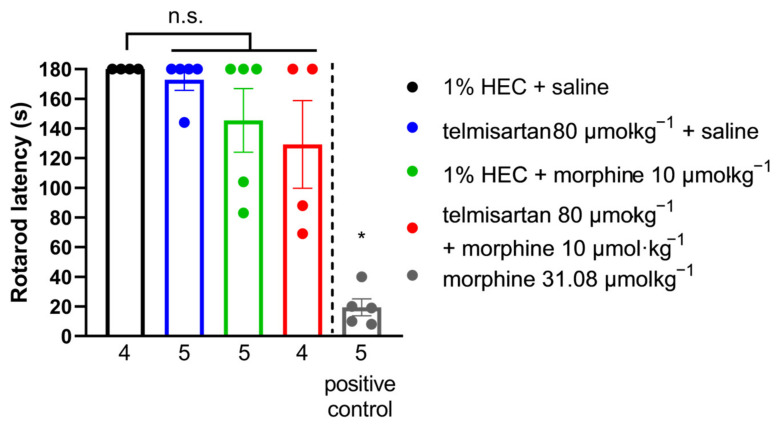
Effect of systemic (oral or subcutaneous) administration of analgesic doses of telmisartan and its combination with morphine on the motor function of naïve animals. Columns represent the time latency of animals in the RotaRod assay ± S.E.M. measured at peak effect of test compounds. Abbreviation “n.s.” stands for not significant. * *p* < 0.05 vs. all other groups, one-way ANOVA followed by Fisher’s LSD post hoc test, *n* = 4–5 per group, exact group sizes are shown in each graph. F (4, 18) = 17.13, *p* < 0.0001.

Telmisartan (80 µmol·kg^−1^ BW, po.), morphine (10 µmol·kg^−1^ BW, sc.) or their combination failed to exhibit motor dysfunction in rats (Figure 6). Morphine treatment (31.08 20 µmol·kg^−1^ BW, sc., as positive control) produced a significant disturbance in motor coordination, seen as shorter fall-off times (latency: 19.4 ± 5.67, *n* = 5 vs. 180 ± 0.00, *n* = 4).

## 3. Discussion

There are many different pharmacotherapeutic approaches for the treatment of neuropathic pain, but none of these has so far proved fully effective. Furthermore, the analgesic effect of current medications is of slow onset, and the side effects are progressively increasing with dose escalation, and thus, limit their further use [5,8]. Therefore, the need to develop novel drugs or new approaches encourages researchers to continue working in this field. In the last decades, many promising drug targets have been identified as potential therapeutic targets for future neuropathic pain treatment, such as angiotensin receptor type 1 [22,23]. The use of combination treatment regimens has also been considered in order to increase the effectiveness without increasing the undesirable effects of the elements of the combination. With respect to the raised issues, we herein present results on the effect of AT1 antagonists, losartan and telmisartan, in a rat neuropathic pain model evoked by pSNL. The antiallodynic effect of these antagonists was then assessed when combined with morphine. Through the present pain assessment model, we have demonstrated that at certain doses both losartan and telmisartan are able to produce significant analgesia in NP after acute oral administration (Figure 1). The pain attenuation effect of losartan or telmisartan is manifested by their ability to ameliorate mechanical allodynia, the hallmark symptom of NP [25]. These results further support previous findings on the analgesic effects of AT1 antagonists in animals with neuropathic pain. There are several studies that demonstrate the analgesic effects of AT1 antagonists in diabetic polyneuropathy [26,27] or chemotherapy-induced neuropathy, among others [28,29]. Indeed, in the peripheral mononeuropathic pain model, previous studies have found that AT1 antagonists such as telmisartan or losartan could attenuate neuropathic pain following chronic treatments in rats that underwent chronic constriction injury (CCI) [30,31]. In this regard, in our present work, we could also demonstrate an analgesic effect for single-dose administration of oral telmisartan or losartan with rat NP evoked by pSNL, but at a dose nearly two-fold higher than in previous studies. In addition, the applied dose range of telmisartan or losartan in previous studies corresponds to the ineffective dose range in our study, tested either after acute or chronic administration. An earlier study by Kim and co-workers showed that CCI of the sciatic nerve, tight ligation of spinal nerves (SNL) and tight partial ligation of the sciatic nerve (pSNL) generally evoked similar time-course behavioral symptoms, but signs of mechanical allodynia were greater in rats subjected to pSNL compared to CCI [32]. At present, the discrepancy in the effect of test compounds regarding the applied dose range can be explained by the type of surgical procedure used to evoke NP in rats (CCI vs. pSNL). Neuropathic pain is a devastating disease evoked by different causes and several underlying pathophysiological mechanisms which have resulted in the existence of multiple therapeutic strategies. Thus, studying the analgesic effects of AT1 blockade is worth doing, although previous work has shown negative results [33]. Indeed, the majority of the findings are promising [26,27,28,29] and our results are in line with these findings, because in the present work, we could achieve an acute antiallodynic effect with losartan and telmisartan. However, the doses administered in the experiments here were high when translated to human doses. It means that losartan and telmisartan were able to produce acute analgesic effect in NP but in doses much higher than those being used in animal studies and clinical practice with respect to analgesia or hypertension, respectively. This observed trend corresponds with findings from our previous studies related to the effect of morphine in diabetic rats with polyneuropathic pain [14] and in the present work regarding mononeuropathic pain (pSNL). In this study, the acute antiallodynic effect of morphine was only achieved in high doses. Therefore, the therapeutic strategy of drug combinations was followed to enhance analgesia and decrease deleterious effects. In this regard, we could prove that the subanalgesic dose of telmisartan in combination with the subanalgesic dose of morphine significantly attenuated NP after chronic treatment and produced an analgesic trend after acute administration (Figure 2). On the other hand, the combination of morphine with losartan failed to show pain attenuation following either acute or chronic administration. The differences between the two ARBs in our study may possibly be explained by their different kinetic or dynamic properties as well. In the current literature, telmisartan is considered to be a drug of particular interest regarding its central RAS-influencing effects [34] because of its highest lipophilicity in the class of ARBs and its penetration capabilities into the CNS [34,35,36,37]. On the other hand, the results of Wang et al. showed that losartan could not induce a significant CNS effect [38]. Furthermore, studies attribute potent neuroprotective effects to telmisartan, explained partly by its AT1 antagonist effect and partly by its unique-in-class peroxisome proliferator-activated receptor-γ (PPAR-γ) agonist effect [39,40]. PPAR-γ agonists have been reported to have relevant suppression on the production of proinflammatory cytokines such as tumor necrosis factor-alpha (TNF-α) and interleukin-1 beta (IL-1β), among others [41]. Peripheral nerve injury-induced microglia activation in the spinal cord results in an increase in these proinflammatory cytokines, which play a profound role in the hyper-excitability and central sensitization that are being seen in neuropathic animals [42,43,44]. In addition, PPAR-γ inhibited the activation of CX3CR1 (fractalkine receptor) in rats developing neuropathic pain [45]. With respect to the impact of losartan on PPAR-γ, to the best of our knowledge, no study has reported on its direct action yet. However, losartan has several metabolites, among them EXP3179 is a minor one, which has been reported to act as an agonist of PPAR-γ [46]. At present, the lack of antiallodynic effect of losartan in combination with morphine can be explained by low levels of EXP3179, its PPAR-γ agonist metabolite, formed from the low dose of losartan in the combination. With respect to repeated morphine administration, it is known to result in increased spinal glial activation as well as the expression of multiple chemokines and cytokines. Thus, the failure of the combination of subanalgesic morphine and losartan to produce analgesia may be due to a small amount of EXP3179 to activate PPAR-γ and, as a consequence, reduced spinal glial inhibition and increased chemokine and cytokine expression, which contribute to the development of opioid analgesic tolerance and allodynia [47].

Fortunately, the promising analgesic results following the combination of telmisartan and morphine could draw our attention to one particular issue, namely the negligible development of analgesic tolerance to morphine when telmisartan was administered simultaneously. Accordingly, further studies were carried out to shed light on the cellular mechanisms beyond the remarkable analgesic effect seen with the combination of telmisartan and morphine. We have performed additional in vitro experiments utilizing the morphine-stimulated [^35^S]GTPγS binding assay to demonstrate the G-protein activating capabilities of morphine in the spinal cord of neuropathic animals treated with morphine or the combination of telmisartan and morphine. The obtained results showed no change in the efficacy (E_max_) of morphine in the spinal samples of either treatment group (Figure 3). However, the salient finding is that in animals receiving telmisartan in combination with morphine for 10 days, morphine retained its potency at the spinal level indicated by the EC_50_ value and attenuated NP evoked by pSNL. On the other hand, 10 days of morphine treatment caused a significant decrease in the potency of the morphine. Previous studies have demonstrated no change in opioid E_max_ values in the spinal cord of mice that develop analgesic tolerance to morphine compared to non-tolerant ones [48]. This is in line with our results; however, we expected a decline in both morphine potency and efficacy in the spinal samples of animals that underwent pSNL, as NP can induce a decrease in the membrane µ-opioid receptor reserve which is reflected by a decrease in the [^35^S]GTPγS binding E_max_ of morphine, as described previously [14,49]. To explain the results shown here with respect to telmisartan, besides its AT1 antagonist effect, the activation of PPAR-γ [39,40] might also be of importance. Activation of PPAR-γ has been reported to delay the development of opioid tolerance [50] and reduced NP [51]. We have further investigated the impact of chronic oral administration of telmisartan on morphine-induced analgesic tolerance in a thermal pain model, the rat tail-flick assay. Our results are in line with data reported previously, regarding PPAR-γ agonists delaying the development of morphine tolerance in mice [50]. PPAR-γ is expressed by neurons, astrocytes, and microglia in the spinal cord, and it can downregulate CXCRs including CXCR4. CXCR4 antagonists have been reported to enhance the morphine analgesic effect [52,53]. CXCRs activate protein kinase C, which in turn phosphorylates µ-opioid receptors, which results in its uncoupling from the G_i_ protein. As a consequence, G protein coupled receptor kinase and arrestin are recruited to the µ-opioid receptor, facilitating its internalization and therefore analgesic-tolerance development [52,54]. These studies proposed a role for a µ-opioid receptor–CXCR4 crosstalk in the spinal cord and the development of opioid tolerance. At present, the mechanism for the measured in vivo and in vitro effects are still not completely understood, and future studies will be needed to elucidate more precisely which factors are beyond analgesic-tolerance control.

Changes in the function of the spinal glutamatergic system have been described in the spinal cord of animals with NP [55,56]. Hence, we assessed the changes in L-glutamate levels in the CSF of rats with NP and treated chronically with subanalgesic doses of telmisartan and morphine separately or in combination. This strategy was also extended to examine the CSF L-glutamate content of rats with developed opioid analgesic tolerance. In these experiments, unexpectedly, the combination of telmisartan and morphine increased L-glutamate content in the CSF of rats showing analgesic response and delayed opioid tolerance. In fact, the mechanism of the observed effect of the combination of telmisartan and morphine related to the increase in glutamate level in CSF is complex and likely involves multiple targets such as AT1, PPAR-γ and opioid receptors which directly or indirectly engage in pain regulation and neuronal protection, among other physiological functions.

The direct action of telmisartan includes an anti-inflammatory effect through activation of PPAR-γ [57] or AT1 inhibition [58], which has been shown in cultured microglia and neurons [39]. Based on these studies, despite the increase in the glutamate content of the CSF in rats with NP and treated with the combination, telmisartan could ameliorate glutamate-induced neuronal-injury-evoked neuropathic pain. It is possible that telmisartan might also protect neuronal cells by decreasing inflammatory response in the spinal cord through either inhibition of AT1 or stimulating PPAR-γ rather than inhibition of glutamate release [39]. Telmisartan has been reported to produce significantly decreased glutamate-induced neuronal injury when applied prior to or concomitantly with glutamate [39]. It is indeed difficult to judge whether neuronal injuries evoked by increased glutamate level upon neuropathy are halted by telmisartan administration. NP and opioid tolerance share overlapping neural changes with respect to glutamate. In this regard, NMDA receptor blockers show an analgesic effect on NP and are able to delay the development of opioid analgesic tolerance [59,60,61,62]. However, it is important to note that the elevated CSF L-glutamate content observed in our experiments does not specifically reflect the function of the dorsal horn of the spinal cord, i.e., the spinal sensory system. It is conceivable that the increased glutamate efflux is a consequence of autonomic or other systems and that it does not act exclusively on NMDA receptors. Thus, future studies are required to understand how the telmisartan and morphine combination can ameliorate NP and delay analgesic tolerance. Finally, we compared the antiallodynic advantage of telmisartan to morphine regarding motor function. Even at the highest-applied analgesic dose of telmisartan alone or in combination with a subanalgesic dose of morphine, there was no measurable motor dysfunction—unlike several current medications used in the treatment of neuropathic pain [63,64].

## 4. Materials and Methods

### 4.1. Animals

Experimental protocols were carried out on male Wistar rats obtained from Toxi-Coop Zrt. (Budapest, Hungary). Animals weighing 120–150 g or 170–200 g at the beginning of the study were used for the neuropathic pain model or the morphine analgesic-tolerance experiments, respectively. A total of 123 animals were used for the neuropathic pain model and 24 animals for the morphine analgesic-tolerance model. Furthermore, 23 animals underwent RotaRod testing for motor coordination. Thus, a total number of 170 rats were used. Animals were maintained under controlled environmental conditions (20 ± 2 °C temperature, 12:12 h light/dark cycle) in standard cages, holding four to five animals per cage in the local animal house of Semmelweis University, Department of Pharmacology and Pharmacotherapy (Budapest, Hungary). Water and standard rodent chow were available ad libitum. Prior to experiments, animals were allowed to acclimatize for at least 1 week. All housing and experiments were performed in accordance with the European Communities Council Directives (2010/63/EU), the Hungarian Act for the Protection of Animals in Research (XXVIII.tv. 32.§) and the local animal care committee (PEI/001/276-4/2013 and PE/EA/619-8/2018).

### 4.2. Materials

Telmisartan and losartan-potassium were obtained from TCI EUROPE N.V. (Zwijndrecht, Belgium), while morphine-HCl was obtained from Alkaloida-ICN (Tiszavasvári, Hungary). Telmisartan was suspended in 1% hydroxyethyl-cellulose solution (HEC), while losartan-potassium and morphine-HCl were dissolved in 0.9% saline. Telmisartan and losartan were administered orally (po.), using stainless steel oral feeding needles (purchased from Animalab Hungary Kft., Vác, Hungary) in a total volume of 5 mL·kg^−1^ bodyweight (BW). Morphine was administered subcutaneously (sc.) in a total volume of 2.5 mL·kg^−1^ BW. Pentobarbital was obtained from Semmelweis University Pharmacy (Budapest, Hungary), and dissolved in 0.9% saline before being administered intraperitoneally (ip.) in a total volume of 2.5 mL·kg^−1^ BW. Diethyl-ether was purchased from Sigma-Aldrich (Budapest, Hungary).

For [^35^S]GTPγS binding assay DMSO, Tris-HCl, EGTA, NaCl, MgCl2 × 6H2O, GDP and the GTP analog GTPγS were purchased from Sigma-Aldrich (Budapest, Hungary). The radiolabeled GTP analog, [^35^S]GTPγS (specific activity: 1250 Ci/mmol, Cat.No.: NEG030H250UC) and the UltimaGoldTM MV aqueous scintillation cocktail was purchased from PerkinElmer (handled by Per-Form Hungaria Kft, Budapest, Hungary).

All compounds were stored and handled as described in the product information sheets.

### 4.3. Experimental Protocols

#### 4.3.1. Mononeuropathic Pain Model

**Figure 7:**
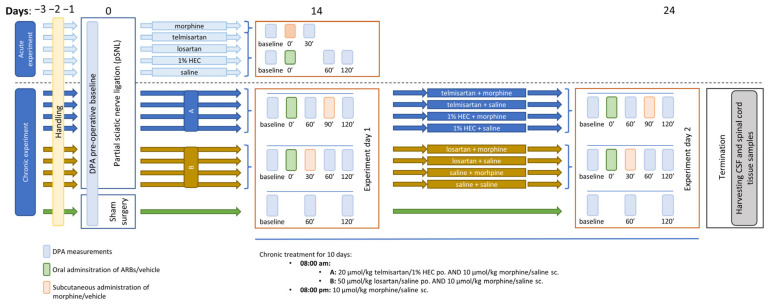
Schematic representation of the study design applied in the mononeuropathic pain model. The figure indicates the timeline of the acute and chronic experiments, involving DPA measurements, pSNL, treatment days and termination.

A schematic summary of the study design of the applied neuropathic pain model is presented in Figure 7. In the days preceding the start of the experiments, handling was performed in order to acclimatize the animals to the experimental conditions. This consisted of placing the animals in the plastic cages of the experimental apparatus once daily. Mechanical allodynia induced by neuropathy was assessed using a dynamic plantar esthesiometer (DPA 37450, Ugo Basile, Italy) as described before [56,65] with the following settings: incrementation: 10 g/s, maximal force: 50 g. The PWTs of animals were measured in grams, following at least five minutes of habituation in the cage in the case of each animal. Each paw was measured three times and the means of the three measurements were used for further analysis. All behavioral studies were performed by the same tester.

First, baseline measurements were performed with DPA to determine the pre-operative PWT. Next, animals underwent partial sciatic nerve ligation (pSNL) based on the method described by Seltzer and colleagues and in our previous studies [56,64,66]. Briefly, under pentobarbital anesthesia (60 mL·kg^−1^ BW), animals were placed on heating pads to maintain 37 °C body temperature. Under aseptic conditions, the right sciatic nerve was exposed without muscle damage at the thigh level. The dorsal ½ of the nerve was tightly ligated with size 7-0 silicon treated silk suture. The wound was closed with two stiches. A separate group of animals used later as controls underwent sham-surgery during which the nerve was exposed without subsequent ligation.

Two weeks after the operation, PWTs of both (operated and intact) hind paws of the animals were determined. Animals were considered neuropathic if there was a 20% decrease in the average *PWT* value of the operated (right) paw compared to the unoperated (left) paw, calculated by the following formula:

(1) $$\frac{{\mathrm{PWT}}_{\mathrm{unoperated}\;\mathrm{paw}}-{\mathrm{PWT}}_{\mathrm{operated}\;\mathrm{paw}}}{{\mathrm{PWT}}_{\mathrm{unoperated}\;\mathrm{paw}}}\times 100$$

Next, randomization was used to allocate animals to control and treatment groups. Following randomization, small adjustments were made where necessary to ensure that the mean baseline PWT values for each group were close to the same. This was carried out to ensure that any subsequent effects of the test compounds were equally well detected. After test compounds or vehicles were administered, the PWTs of animals were determined again at 60 and 120 min as depicted in Figure 1. A group of animals was used to determine the acute antiallodynic effect of different doses of telmisartan (20, 40 and 80 µmol·kg^−1^ BW, po.), losartan (50, 100 and 150 µmol·kg^−1^ BW, po.) and morphine (10 and 20 µmol·kg^−1^ BW, sc.). In experiments aiming to assess the effect of the combination of ARBs and morphine, the compounds were administered in a time-shifted manner (ARBs at 0 min and morphine at 30 or 90 min) so that the peak effect of the combination elements always coincided in time. The time intervals were chosen according to the data obtained in the first part of the study, i.e., the acute experiments with different doses of the two ARBs. Based on our previous studies, the peak effect of morphine was expected at 30 min after subcutaneous administration [14,24].

Another group of animals was subjected to chronic treatment with a combination of morphine and ARBs, using doses that were found to be subanalgesic in acute tests (20, 50 and 10 µmol·kg^−1^ BW for telmisartan, losartan and morphine, respectively). In this group, morphine was administered subcutaneously twice a day, while ARBs were administered orally once a day. On the 24th day following Seltzer surgery, another set of DPA measurements were carried out on chronically treated animals. Following this, animals were sacrificed by diethyl-ether overdose after which spinal cord tissue and CSF samples were obtained for further in vitro analyses.

#### 4.3.2. Morphine Analgesic-Tolerance Model

**Figure 8:**
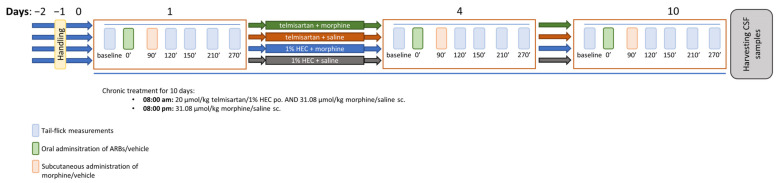
Schematic representation of the study design applied in the morphine analgesic-tolerance model. The figure indicates the timeline of the tail-flick measurements treatment days and termination.

A schematic summary of the applied opioid analgesic-tolerance protocol is presented in Figure 8. In the days preceding the start of the experiments, handling was performed in order to acclimatize the animals to the experimental conditions. This consisted of placing the animals in the tail-flick apparatus with blindfolds on, once daily. Acute thermal pain sensation was assessed using radiant heat tail-flick test (IITC Life Science, Woodland Hills, CA, USA) as described previously [67,68] with some modifications. Briefly, light-intensity was adjusted to set the control tail-flick latency under 4 s. Cut-off time was set to 8 s to avoid tissue damage. A baseline latency was measured before and after test compound or vehicle administration at the depicted time points (see Figure 2). All behavioral studies were performed by the same tester.

Animals were randomized into groups and rendered tolerant to morphine by subcutaneous injections of 10 mg·kg^−1^ or 31.08 µmol·kg^−1^ BW morphine twice daily (8 a.m. and 8 p.m.) for 10 days. Saline injections (2.5 mL·kg^−1^ twice daily) were used in the control animals. In addition to morphine or saline, the animals also received telmisartan (20 µmol·kg^−1^, po.) or 1% HEC (5 mL·kg^−1^, po.) once daily (8 a.m.). The degree of analgesic-tolerance development was determined using the tail flick test on days 4 and 10. Following this, animals were sacrificed by diethyl-ether overdose and cerebrospinal fluid (CSF) samples were obtained for further in vitro analyses.

#### 4.3.3. Morphine-Stimulated [^35^S]GTPγS Binding Assay

Rats were decapitated and their spinal cords were quickly removed. The spinal cords were prepared for membrane preparation for the [^35^S]GTPγS binding experiments, as described previously [56]. Briefly, spinal cord samples were homogenized in ice-cold TEM buffer composed of 50 mM Tris-HCl, 1 mM EGTA, 3 mM MgCl2, and 100 mM NaCl, pH 7.4 with a Teflon-glass homogenizer. The homogenate was centrifuged at 18,000 rpm for 20 min at 4 °C. The resulting supernatant was discarded, and the pellet was further incubated at 37 °C for 30 min in a shaking water-bath. Then, centrifugation was repeated as described above. The final pellet was suspended in ice-cold TEM buffer and stored at −80 °C. The protein content of the membrane preparation was determined by the method of Bradford, BSA being used as a standard [69].

In the [^35^S]GTPγS binding experiments, we measured the GDP → GTP exchange of the Gαi/o protein in the presence of a given ligand. The nucleotide exchange was monitored by a radioactive, non-hydrolyzable GTP analogue, [^35^S]GTPγS. The functional [^35^S]GTPγS binding experiments were performed as previously described [48,49], with modifications. Briefly, the membrane homogenates were incubated at 30 °C for 60 min in TEM buffer containing 20 MBq/0.05 mL [^35^S]GTPγS (0.05 nM) and 0.1–10 µM concentrations of the GlyT inhibitors (alone or in combination) and DAMGO. The experiments were performed in the presence of excess GDP (30 µM) in a final volume of 1 mL. Total binding was measured in the absence of test compounds, non-specific binding was determined in the presence of 10 µM unlabeled GTPγS and subtracted from total binding. The difference represents basal activity. The reaction was terminated by rapid filtration under vacuum (Brandel M24R Cell Harvester, Gathersburg, MD, USA), and washed three times with 5 mL ice-cold 50 mM Tris-HCl (pH 7.4) buffer through Whatman GF/B glass fibers. The radioactivity of the filters was detected in UltimaGoldTM MV aqueous scintillation cocktail with Packard Tricarb 2300TR liquid scintillation counter (Per-Form Kft, Budapest, Hungary). [^35^S]GTPγS binding experiments were performed in triplicate and repeated at least three times.

#### 4.3.4. Capillary Electrophoresis Analysis of Glutamate Content

L-glutamate content of cerebrospinal fluid (CSF) samples was measured by the capillary electrophoresis laser-induced fluorescence method carried out as described previously [56,70].

Animals in our mononeuropathic pain model or morphine analgesic-tolerance model were sacrificed after 10 days of treatment as discussed above. CSF samples were obtained by cisterna magna puncture and centrifuged at 2000× *g*, at 4 °C for 10 min. Samples were frozen immediately and stored at −80 °C until further processing. On the day of the experiment, samples were deproteinized by mixing with 2 volumes of pure acetonitrile and centrifuged at 20,000× *g* for 10 min at 4 °C. Supernatants were collected, diluted five times with acetonitrile-distilled water solution (2:1; *v*/*v*) and subjected to derivatization with 7-fluoro-4-nitro-2,1,3-benzoxadiazole (NBD-F) (1 mg/mL final concentration) in 20 mM borate buffer pH 8.5 for 20 min at 65 °C. Five µM L-cysteic acid was used as internal standard.

Derivatized samples were analyzed using a P/ACE MDQ Plus capillary electrophoresis system coupled with a laser-induced fluorescence detector equipped with a laser source of excitation and emission wavelengths of 488 and 520 nm, respectively, (SCIEX, Framingham, MA, USA). Separations were carried out in polyacrylamide-coated fused silica capillaries (i.d.: 75 µm, effective/total length: 50/60 cm) using 50 mM HEPES buffer pH 7.0 containing 6 mM hydroxypropyl amino-β-cyclodextrin at 15 °C by applying −30 kV constant voltage.

#### 4.3.5. Motor Function Testing

The effect of telmisartan, morphine and their combination on the motor function of animals was assessed using the rotarod assay (Rat RotaRod, model 7750, Ugo Basile, Italy). On the day preceding the experiment, animals were trained to stay on the rotating rod of the instrument. Rotation speed was set at 16 rotations per minute (RPM) and cut-off time was set at 180 s. On the day of the experiment, the animals were treated orally with the highest tested dose of telmisartan (80 µmol·kg^−1^), the dose of morphine used in the combination experiments (10 µmol·kg^−1^), their combination or their vehicles (1% HEC or saline, respectively). The motor coordination of animals was tested at the time of peak effect of test compounds. The compounds were administered in a time-shifted manner (ARBs at 0 min and morphine at 90 min) so that the peak effect of the combination elements coincided in time. Latency on the rotarod instrument was noted in s (fall-off time). High dose morphine (31.08 µmol·kg^−1^) was used as positive control.

### 4.4. Statistical Analysis

All values are presented as mean ± standard error of means (S.E.M.). The statistical analysis was performed using GraphPad Prism software (version 8.0.1; GraphPad Software Inc., San Diego, CA, USA). *p* < 0.05 was considered statistically significant. Two-way ANOVA followed by Tukey’s post hoc test was used for multiple comparisons between related groups. One-way ANOVA followed by Fisher’s LSD post hoc test was used to compare independent groups. The post hoc tests were conducted only if F in ANOVA achieved *p* < 0.05. ROUT analysis was performed to identify outliers, with Q value = 0.5%.

## 5. Conclusions

AT1 antagonists attenuated mononeuropathic pain after acute treatment. A subanalgesic dose of telmisartan in combination with a subanalgesic dose of morphine proved to be effective against NP and morphine analgesic tolerance. Concomitantly, telmisartan administration also restored morphine potency in neuropathic rats and delayed morphine tolerance. Thus, in conditions with a loss of opioid efficacy, such as neuropathic pain or the development of opioid analgesic tolerance, telmisartan may make adequate pain control achievable. This can be carried out by finding entirely new pharmacological targets, or by exploiting multitarget treatment options that improve the efficacy of the available agents. These findings may provide the preclinical basis for the use of telmisartan in pain conditions with opioid impairment and raise the possibility of repurposing angiotensin receptor blockers in NP or opioid analgesic tolerance.

## Figures and Tables

**Table 1:** Morphine-stimulated [^35^S]GTPγS binding data on spinal cords obtained from animals undergoing 10 days of chronic treatment. EC50 and Emax values were calculated individually for each animal sample and means ± S.E.M. are presented here.

Treatment Group (10 Days of Treatment)	GTPγS Binding	
	EC50 ± S.E.M. (nM)	Emax ± S.E.M. (%)
1% HEC + saline (*n* = 5)	217.2 ± 137.8	134.1 ± 6.8
1% HEC + morphine (*n* = 5)	1864.0 ± 783.5 *^δ^	140.2 ± 3.4
telmisartan + morphine (*n* = 5)	285.0 ± 125.1	136.5 ± 1.8

* *p* < 0.05 vs. telmisartan + morphine; ^δ^ *p* < 0.05 vs. control, one-way ANOVA followed by Fisher’s LSD post hoc test, *n* = 5 per group.

**Figure A1:**
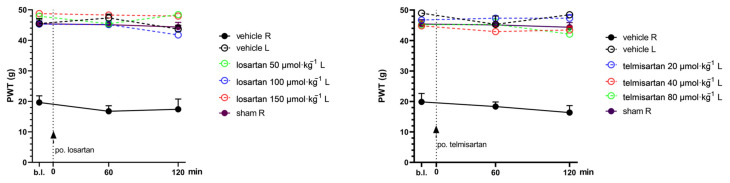
PWT values of the left (intact) paws of animals treated with losartan or telmisartan or their vehicles in the acute experiments. Graphs show the means of PWT ± S.E.M. in grams, after the pSNL in the indicated time points and treatment groups.

**Figure A2:**
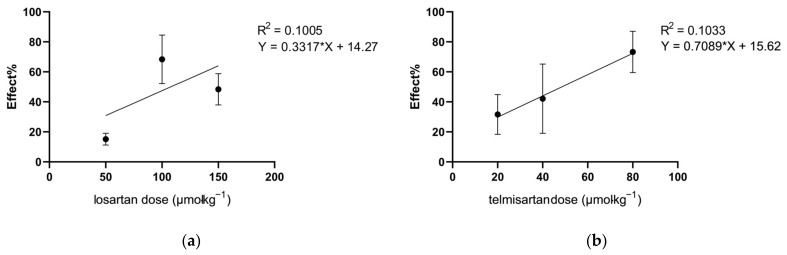
Dose–response curves of losartan (**a**) and telmisartan (**b**) in mononeuropathic animals obtained with DPA. Antinociceptive effect (%) was calculated at peak effect (60 or 120 min for losartan and telmisartan, respectively) as $({\mathrm{PWT}}_{\mathrm{peak}\;\mathrm{effect}}/{\mathrm{PWT}}_{\mathrm{baseline}})\times 100-100$. ED_50_ values calculated from the linear regression are 107.7 and 48.5 µmol·kg^−1^ BW for losartan and telmisartan, respectively. Graphs show the means of the antinociceptive effect ± S.E.M.

## Data Availability

The data that support the findings of this study are available from the corresponding author upon reasonable request.
